# Reappraisal of Alternative Treatments for Non-melanoma Skin Cancers on YouTube: A Cross-Sectional Analysis

**DOI:** 10.7759/cureus.90522

**Published:** 2025-08-19

**Authors:** Udokama Ezekwe, Andrea Quartey, Sabrina Zheng, Amor Khachemoune

**Affiliations:** 1 Dermatology, Lewis Katz School of Medicine, Temple University, Philadelphia, USA; 2 Dermatology, Paul L. Foster School of Medicine, Texas Tech University, El Paso, USA; 3 Dermatology, Premier Dermatology, Ashburn, USA; 4 Dermatology, Istanbul Medipol University, International School of Medicine, Istanbul, TUR; 5 Dermatology, CuraPep, LLC, Garden City, USA

**Keywords:** alternative medicine, basal cell carcinoma, homeopathic remedies, skin cancer, social media, squamous cell carcinoma

## Abstract

Background and objective: Social media plays a significant role in patient education as many US Internet users obtain health information online. YouTube is a popular search engine among people looking for dermatologic advice. Our study assesses the content on homeopathic remedies for non-melanoma skin cancers (NMSCs) available on YouTube.

Methods: We searched YouTube in a private browsing tab for “natural skin cancer remedies,” “alternative skin cancer treatment,” and “holistic skin cancer treatment” in separate searches. The top 40 videos meeting inclusion criteria in each search were analyzed. For data extraction, the video characteristics, engagement metrics and content themes were recorded. Duplicates were removed.

Results: 61 videos were analyzed in total. 22 (36.1%) of the videos were created by homeopathic YouTube channels, 20 (32.8%) by laypeople and/or influencers, 11 (18%) by physicians and pharmacists, 4 (6.7%) by news channels, and 4 (6.7%) by other personnel. The 10 most frequently mentioned remedies included green tea (n=12, 19.7%), turmeric/curcumin (n=12, 19.7%), coconut oil (n=9, 14.8%), black salve (n=7, 11.5%), apple cider vinegar (n=7, 11.5%), baking soda (n=7, 11.5%), garlic (n=7, 11.5%), frankincense oil (n=6, 9.8%), eggplant (n=6, 9.8%), and milk thistle (n=6, 9.8%).

Discussion: Although most videos were created by homeopathic channels, they had the lowest engagement. Videos created by healthcare professionals achieved significantly higher engagement. Thus, even when seeking natural remedies on social media, viewers prefer content created by professionals. There is some existing literature on the role the recommended remedies play in preventing and/or treating skin cancers.

Conclusion: Dermatologists should be aware of the various at-home therapies patients may try for their skin cancer. They should consider creating their own reliable and accurate social media content to educate the public about the risks of these dangerous trends and emphasize the importance of seeking evaluation for suspicious skin lesions.

## Introduction

Basal cell carcinoma (BCC) and squamous cell carcinoma (SCC) are the most common non-melanoma skin cancers (NMSCs), with incidence rates rising globally [1]. While NMSC treatment is almost always lesion removal, current guidelines recommend the type of treatment based on patient factors and lesion characteristics. These treatment methods include Mohs micrographic surgery (MMS), standard surgical excision, electrodesiccation and curettage (EDC), radiation therapy, photodynamic therapy, cryosurgery, topical therapies, and systemic medications [1].

Social media has become a widely used source of medical information; however, it carries the risk of disseminating misinformation. Patients can find information on NMSC treatment on Internet platforms like YouTube promoting alternative and homeopathic treatments for skin cancer, though their credibility remains uncertain [2]. This may potentially influence patient decision-making and care-seeking behaviors [3].

This study analyzes the content of YouTube videos discussing homeopathic remedies for NMSC, evaluating the types of treatment sources, and audience engagement. By assessing the prevalence and credibility of these alternative treatment claims, we aim to highlight the potential risks associated with misinformation in NMSC treatment and its effects on patients' decision-making. Given the growing number of patients who use social media as a source of health information, analyzing YouTube content is clinically relevant. The misinformation on a popular social media platform such as YouTube can influence patient perceptions of disease, delay diagnosis and diminish adherence to evidence-based dermatologic care.

## Materials and methods

Search strategy

In March 2025, we searched YouTube by relevance in a private browsing tab for “natural skin cancer remedies,” “alternative skin cancer treatment,” and “holistic skin cancer treatment” in separate searches. The top 40 videos relevant to each search item were recorded.

YouTube was selected as the sole platform for analysis due to its popularity among individuals seeking health-related information. Other platforms such as TikTok and Instagram were excluded in order to maintain consistency in video format, search functionality, and available engagement metrics.

Inclusion and exclusion criteria

Inclusion criteria included English-only videos that specifically mentioned skin cancer and discussed treatments or remedies that were described as natural, organic, holistic, or homeopathic. For the purposes of this study, such remedies were defined as treatments from non-pharmaceutical, plant-based, or alternative medicine sources that are not part of standard dermatologic care. No minimum video duration was required for inclusion.

Exclusion criteria encompassed videos that were not in English, those addressing skin cancer in animals, content that did not explicitly pertain to skin cancer, and videos that predominantly discussed melanoma. Additionally, videos emphasizing conventional treatments, including MMS, pharmacologic interventions, laser therapy, and radiation, were excluded. Duplicate videos within the three search results were also removed. They were identified based on title and URL and only counted once in the final dataset. Only publicly available videos were included in analysis. Private, removed, and age-restricted content were excluded.

Data extraction

For data extraction, the following variables were recorded for each video: creator type (e.g. physician, layperson, influencer, homeopathic channel, news channel), year of upload, video length, and engagement metrics (number of views, likes, dislikes, and comments). The term "homeopathic channels" was used to describe YouTube channels that did not feature people in their videos and were dedicated to the use of natural substances, such as herbs and other plants, to treat medical conditions. Content-related variables included the recommended remedies, the type of skin cancer discussed (cutaneous squamous cell carcinoma (cSCC), BCC, or generic skin cancer), the presence of a medical disclaimer, and whether the video indicated that the patient had been evaluated by a physician. Each video was categorized as either patient education or sharing of patient experiences/testimonies, as well as whether the video was aimed at treatment or prevention.

Statistical analysis

Descriptive statistics were used to summarize video characteristics, engagement metrics, and content themes. Engagement metrics were analyzed to assess correlations with creator type and year of upload.

## Results

A total of 61 videos uploaded between 2008 and 2024 were analyzed. Collectively, the videos were viewed more than 6.8 million times (range: 8-4,135,703 views). Majority of the videos were created by homeopathic YouTube channels (n=22, 36.1%), followed by laypeople and/or influencers (n=20, 32.8%), physicians and pharmacists (n=11, 18.0%), news channels (n=4, 6.7%), and other creators, including estheticians and businesses specializing in supplements or herbal products (n=4, 6.7%). The average video length was 8 minutes and 19 seconds (range: 37 seconds-77 minutes and 48 seconds). The 61 videos analyzed are included in Appendix 1.

The 10 most frequently mentioned remedies included green tea (n=12, 19.7%), turmeric/curcumin (n=12, 19.7%), coconut oil (n=9, 14.8%), black salve (n=7, 11.5%), apple cider vinegar (n=7, 11.5%), baking soda (n=7, 11.5%), garlic (n=7, 11.5%), frankincense oil (n=6, 9.8%), eggplant (n=6, 9.8%), and milk thistle (n=6, 9.8%). Other remedies recommended included various vitamins, aloe vera, omega-3 in fish oil, black raspberry seed oil, sweet potato, pomegranate, avocado, ginkgo, lemon, red wine, strawberry, echinacea, leafy green vegetables, nuts, beans, euphorbia peplus/petty spurge/cancer weed, hemp seed oil, grapefruit, oranges, cat’s claw/uncaria tomentosa, myrrh oil, honey, white tea, apigenin, olive oil, sunflower oil, sesame oil, peanut oil, tomato paste, cocoa, grapes, onion, soy, ginger, saffron, pepper, oregano, flaxseed, astragalus, parsley, cumin, castor oil, beets, butternut squash, sage, red clover, chia seeds, cannabidiol oil, propolis, bee venom, white vinegar, and radium weed. A summary of the top 10 treatments, their active ingredients, and existing literature on their use for skin cancer is presented in Table 1.

**Table 1:** Prevalence and scientific evidence of YouTube-recommended alternative skin cancer treatments GTP: Green tea polyphenol; UVB: Ultraviolet B radiation; NHEK: Normal human epidermal keratinocyte; DAS: Diallyl sulfide; DADS: Diallyl disulfide; DATS: Diallyl trisulfide; BCC: Basal cell carcinoma; SCC: Squamous cell carcinoma; DHS: 2,3-dehydrosilibinin

Natural Remedy	Videos Appeared In n (%)	Active Components	Evidence for Skin Cancer Use	Study
Green Tea	12 (19.70%)	Epigallocatechin gallate	Mantena et al. investigated the mechanism by which GTPs, also known as catechins, prevent UVB-induced skin cancer in mice. Results showed that oral administration of GTPs resulted in significant protection against photocarcinogenesis in terms of tumor incidence, tumor multiplicity, and tumor growth.	Mantena et al. [4]
Turmeric	12 (19.70%)	Curcumin	Jose et al. evaluated the effectiveness of iontophoretic co-delivery of curcumin and anti-STAT3 siRNA using liposomes against skin cancer. The in vivo efficacy studies were performed in a mouse model of melanoma skin cancer. Co-administration of the curcumin and STAT siRNA using liposomes significantly (p<0.05) inhibited the tumor progression compared with either liposomal curcumin or STAT3 siRNA alone.	Jose et al. [5]
Coconut Oil	9 (14.80%)	Lauric acid	No supporting evidence found.	
Black Salve	7 (11.50%)	Zinc chloride and sanguinarine from bloodroot	A preclinical study compared the antiproliferative and apoptotic potential of sanguinarine against human epidermoid carcinoma (A431) cells and NHEKs. The study concluded that sanguinarine induced a cell growth-inhibitory response at micromolar concentrations via apoptosis in human A431 squamous carcinoma cells. However, NHEKs died via necrosis at much higher doses of sanguinarine.	Ahmad et al. [6]
Apple Cider Vinegar	7 (11.50%)	Acetic acid	No supporting evidence found.	
Baking Soda	7 (11.50%)	Sodium bicarbonate	No supporting evidence found.	
Garlic	7 (11.50%)	Allicin	Wang et al. investigated the efficacy and underlying mechanisms of DAS, DADS, and DATS, organosulfur compounds derived from garlic, in the treatment of skin cancer using both in vitro and in vivo models. The study demonstrated that DATS exhibited superior growth inhibition of human melanoma (A375) and BCC cell lines compared to DAS and DADS.	Wang et al. [7]
Frankincense Oil	6 (9.80%)	Boswellic acid	A case report by Fung et al. reported a case of a 56-year-old man with BCC on the arm and chest, which were both treated with frankincense oil for 4 months. Upon completion of therapies, biopsies confirmed complete resolution of the BCC on the arm, but focal residual BCC on the chest.	Fung et al. [8]
Eggplant Extract	6 (9.80%)	Solasodine glycosides	In an open-label study conducted in Australia, a topical formulation containing solasodine glycosides (Curaderm) was applied to 86 patients, including 39 with BCC and 29 with SCC. Complete lesion regression was confirmed by punch biopsy in 100% of cases at a three-month follow-up. In contrast, a placebo formulation demonstrated no therapeutic effect on a smaller subset of treated lesions.	Cham et al.[9]
Milk Thistle	6 (9.80%)	Silymarin	Tilley et al. investigated the efficacy and underlying mechanisms of silibinin, a crude extract from silymarin, and its oxidation product, DHS, in the treatment of BCC using both in vitro and in vivo models. The study demonstrated that silibinin induced apoptosis in murine BCC cell lines in vitro and inhibited BCC tumor growth in a mouse model by suppressing mitogenic signaling pathways.	Tilley et al. [10]

The majority of videos (n=47, 77.1%) did not specify the type of skin cancer targeted by the treatment, instead using the general term "skin cancer." A total of 10 (16.4%) of the videos specifically addressed BCC, while six (9.8%) focused on SCC. Medical disclaimers were present in only 21 (34.4%) of the videos, and just 10 (16.4%) included patients who explicitly stated they had consulted a physician. The majority of videos (n=48, 78.7%) were intended for patient education, while 13 (21.3%) represented patient experiences or testimonials. Regarding the purpose of the ingredients discussed, 38 (62.3%) of the videos focused on treatment, 12 (19.7%) on prevention, and eight (13.1%) addressed both treatment and prevention.

**Figure 1:**
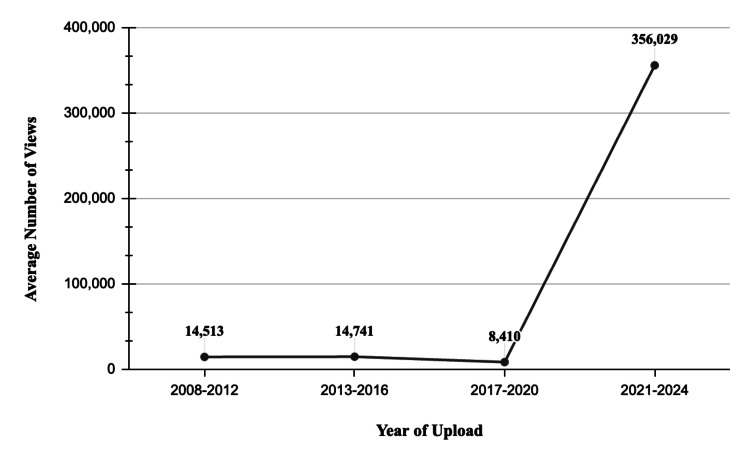
Line graph showing the average number of views per video between the years 2008 and 2024

**Figure 2:**
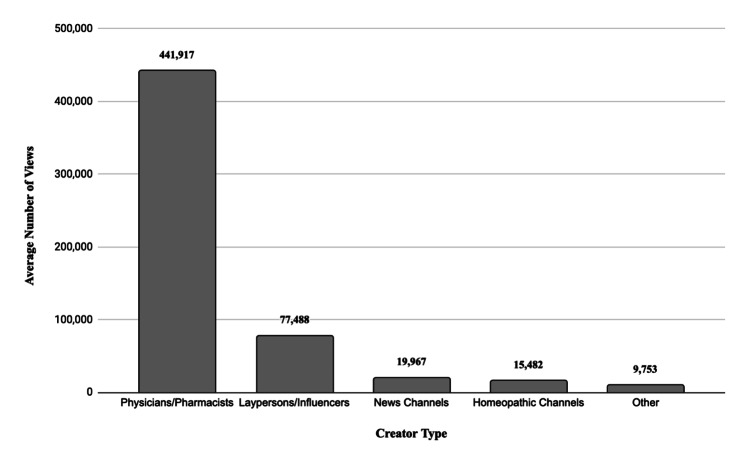
Bar graph showing the average number of views per video according to creator type

Engagement metrics demonstrated significant variation by creator type and year. Videos uploaded between 2021 and 2024 exhibited the highest average number of views, likes, and comments compared to videos from earlier periods. Additionally, videos created by physicians or pharmacists achieved markedly higher engagement metrics compared to those produced by other creator types. The exact figures are illustrated in the Figures 1, 2.

## Discussion

The present study reviewed and quantified unconventional treatment recommendations for NMSCs available on YouTube, with the primary aim of understanding the types of therapies patients may attempt at home, identifying the creators of this online content, and evaluating viewer engagement with these videos. Although the majority of videos on this topic were created by homeopathic YouTube channels (n=22, 36.1%), these videos had the lowest engagement, averaging 15,482 views per video. In contrast, videos created by certified healthcare professionals, such as physicians and pharmacists, achieved significantly higher engagement, with an average of 441,917 views per video - over 28 times greater. This finding suggests that even when seeking natural remedies on social media, viewers demonstrate a preference for content created by healthcare professionals.

The most recommended homeopathic treatments for skin cancer in the reviewed videos were topical turmeric and green tea consumption. There is some existing literature on the role these two agents play in preventing and/or treating skin cancers. Turmeric contains the active compound curcumin, which has demonstrated the ability to inhibit melanoma cell migration and invasion in vitro. It induces apoptosis by activating caspases three and eight, downregulates the JAK-2/STAT3 signaling pathway, and inhibits the AKT/mTOR pathway [11,12]. Two articles by Jose et al. demonstrated that curcumin, when encapsulated in cationic liposomes and complexed with STAT3 siRNA, effectively inhibited human SCC cell growth in vitro and suppressed melanoma growth in both in vitro and in vivo models [5,13]. As for green tea, its active component epigallocatechin-3-gallate inhibits β-catenin signaling within the Wnt pathway, thereby decreasing the risk of tumor development [12]. Furthermore, a study by Mantena et al. found that that oral administration of green tea polyphenols (GTPs) in the drinking water of mice resulted in significant protection against the development of NMSC [4]. Although these preclinical studies are promising, there are currently no clinical trials demonstrating the efficacy of turmeric or green tea compounds in the treatment of NMSC in humans. Therefore, the evidence supporting these herbal preparations is not sufficiently robust to serve as a reliable alternative to current standard therapies [11].

While a few studies have analyzed videos related to skin cancer on YouTube, this is the first study to specifically evaluate the content on homeopathic remedies for NMSCs available on the platform [14-16]. Consistent with findings from previous research on this topic, our study revealed that many videos provide misleading information, potentially discouraging viewers from seeking appropriate dermatologic care for their skin cancer [17]. Notably, we found that numerous video creators promote at-home and "natural" remedies as superior to treatments developed through years of scientific research, potentially influencing patient decision-making.

By analyzing the videos and accompanying comment sections included in this study, we gained insight into the perspectives and reasoning of some patients who opt for unconventional treatment methods. Multiple creators, particularly those categorized as laypersons, mentioned financial constraints as a primary motivation for resorting to natural remedies. One video featured a woman who after demonstrating how she prepared a concoction of baking soda, apple cider vinegar, and coconut oil stated, “This will just have to work for now since no one is taking my insurance.” Aside from the financial component, many creators emphasized the belief that natural substances can cure any ailment without the unwanted side effects that accompany conventional medications. This was demonstrated in a video by a homeopathic channel that recommended eggplant, frankincense, and black raspberries. The narrator prefaces the video with the following statement: “You may think painful laser treatments are the best way to get rid of skin cancers. Or that dangerous radiation or chemo are the answers… But there are harmless natural solutions hiding in places you’d least expect. Here are five secret ways to fight skin cancer.”

We also found variety in the qualitative content amongst the different creators. Laypersons and influencers often presented testimonies in a matter-of-fact manner that their home remedy had in fact cured their skin cancer. For example, a layperson made a video with her friend who had been diagnosed with a BCC. In the video, her friend stated: “I treated a spot on my leg that the doctors thought was BCC with baking soda and coconut oil.” She went on to talk about another spot that occurred on her face that she was currently treating. While homeopathic channels usually did not give specific anecdotes but spoke generally about ingredients that could be used to treat skin cancer. For instance, one made the following statement about eggplant: “Purple eggplant has been clinically demonstrated as a compelling treatment for keratoses, BCCs and SCCs.” Videos made by health care providers on the other hand, were usually either directly debunking dangerous trends such as black salve or discussing ingredients that can help prevent skin cancer but not necessarily treat it. An example of this was a video made by a board-certified emergency medicine physician who stated, “These are some skin cancer fighting foods that everyone should eat!” He mentioned red wine, sweet potato, pomegranates, strawberries, avocado, and salmon as food/drinks that help with the prevention of sunburns and skin cancer.

The popularization of homeopathic remedies for skin cancer poses significant risks to patients. Beyond delaying timely diagnosis and appropriate management, thereby increasing the risk of disease progression and mortality, certain alternative treatments may directly exacerbate the patient’s condition. One such example is the topical escharotic agent black salve, which contains varying amounts of sanguinarine and zinc chloride. Black salve has regained popularity as a natural skin cancer treatment in recent years. While a few case reports suggest clearance of the primary lesion, many document recurrence with advanced cancer and massive scarring, and at least one case of BCC led to bone metastasis and the patient's death [18].

A potential solution to this problem is the creation of social media content by dermatologists and other physicians aimed at exposing the dangers of these unconventional treatments. For instance, some videos included in this study were created by physicians who debunked black salve as a skin cancer remedy. These videos highlighted cases of patients who used black salve and subsequently experienced severe tissue necrosis and massive scarring, necessitating plastic reconstructive surgery to address the resulting damage. Furthermore, videos created by healthcare professionals consistently promoted evidence-based management of NMSCs and emphasized the importance of early detection. They included medical disclaimers, cited scientific references and encouraged viewers to seek evaluation from a board-certified dermatologist. These videos have significantly higher engagement metrics than other creator types, demonstrating that viewers value credible and professional medical voices.

Additionally, it is important for viewers to know how to critically assess online content for its validity. They should be cautious of several warning signs indicating non-legitimate or potentially harmful information. These include videos that do not provide medical disclaimers or fail to advise viewers to consult a licensed healthcare professional. Furthermore, videos that use specific “clickbait” language to promote their remedies as “miracle cures” or “100% natural alternative” are likely to be non-legitimate. Viewers should also be wary of content that directly discredits evidence-based medicine and promotes the notion that “natural is better.” Rather, they should prioritize content created by certified professionals who provide information backed up by scientific research and further stress the importance of patients making a healthcare appointment to be seen by a provider. Finally, viewers are encouraged to go as far as confirming the credentials of the content creators who identify as healthcare professionals via a quick Google search. Awareness of these warning signs and strategies can empower patients to better discern misleading content sources and avoid poor outcomes.

A notable strength of this study is its comprehensive analysis of a large dataset, incorporating detailed metrics on video engagement and content characteristics. However, the study has several limitations. First, the restriction to English-language videos excluded potentially relevant content in other languages. Second, despite using a private browsing tab, the possibility of selection bias introduced by the YouTube algorithm cannot be entirely ruled out. Additionally, the exclusion of shorter video formats, such as YouTube Shorts, represents an additional limitation, as these may also contain relevant information. In addition, while we systematically evaluated the accuracy of content, the assessment of misinformation was qualitative in nature and based on the presence or absence of evidence-based claims rather than a formalized scoring rubric. Future studies could employ standardized misinformation assessment tools to allow for more granular analysis.

Finally, we would like to reiterate that while several natural remedies mentioned in the analyzed videos have been found to have anticancer activity in preclinical studies, these findings are limited to in vitro or animal models and cannot be generalized to human treatment without robust clinical trials. None of these agents have been proven to match or exceed the effectiveness of the standard treatments for NMSC. Thus, they should not be considered substitutes.

## Conclusions

The rise of social media as a source of health information has led patients to seek guidance from platforms such as YouTube regarding alternative and homeopathic treatments for dermatologic conditions including NMSCs. A wide variety of natural remedies are promoted on this platform such as green tea, turmeric/curcumin, coconut oil, black salve, apple cider vinegar, baking soda, garlic, frankincense oil, eggplant, and milk thistle. While some literature suggests potential anticancer properties of these natural ingredients, there is insufficient evidence to support their efficacy as primary treatments for skin cancer when compared to the current standard of care. Dermatologists should be aware of the various at-home therapies patients may try for their skin cancer as well as their impact. Furthermore, they should consider creating their own reliable and accurate social media content to educate the public about the risks of these dangerous trends. Emphasizing the importance of seeking professional evaluation - and, if necessary, treatment - from a board-certified dermatologist for any suspicious skin lesions can help to prevent harmful self-treatment that may lead to advanced disease, increased morbidity, and even mortality from a condition that is otherwise highly treatable when managed with evidence-based care.

## Appendices

Appendix 1

**Table 2:** List of YouTube videos analyzed

No.	Video Title	URL
1	Fighting skin cancer with tea	https://www.youtube.com/watch?v=NmVfu6v-7nU&pp=ygUcbmF0dXJhbCBza2luIGNhbmNlciByZW1lZGllcw%3D%3D
2	4 Remedies For Sunburn | Natural Remedies | Skin Cancer | natural cure | sunburn remedies	https://www.youtube.com/watch?v=qZRZ4c4yCD4
3	Doctors Warn 'Black Salve' Cure For Skin Cancer Is More Ruse Than Remedy	https://www.youtube.com/watch?v=JYLelEmbnvU&pp=ygUcbmF0dXJhbCBza2luIGNhbmNlciByZW1lZGllcw%3D%3D
4	4 Home Remedies For Skin Cancer	https://www.youtube.com/watch?v=IibJKCj0Bi4&pp=ygUcbmF0dXJhbCBza2luIGNhbmNlciByZW1lZGllcw%3D%3D
5	Herbal Remedy that Prevent Skin Cancer and PhotoAging	https://www.youtube.com/watch?v=nQx77z042BE
6	Proven Natural Remedies for Skin Cancer	https://www.youtube.com/watch?v=XXV2c98914w
7	Black Salve as an Alternative Cancer Cure	https://www.youtube.com/watch?v=VbO5RqJCywo
8	A Give Away of One Of My Favorites and How I Naturally Cured My Basal Cell Skin Cancer	https://www.youtube.com/watch?v=N33KcPmnPME
9	7 Natural Remedies for Skin Cancer	https://www.youtube.com/watch?v=0frfwo3Cqvc
10	Best Oils to Prevent Skin Cancer	https://www.youtube.com/watch?v=WhQj6AdgrL8
11	She Tried a DIY Treatment For Skin Cancer - BLACK SALVE! - Plastic Surgeon Reacts to BOTCHED!	https://www.youtube.com/watch?v=rtzxTEoNweg
12	Diet and skin cancer prevention (and younger looking skin too!)	https://www.youtube.com/watch?v=cqvmt6bYWFI&t=83s
13	Top 8 Herbs That PREVENT And KILL Cancer!	https://www.youtube.com/watch?v=NjbmGNJiF7s
14	Cancer Dies When You Eat These 12 Foods (Cancer SECRETS)	https://www.youtube.com/watch?v=WGbFnp56csg
15	DIY Cancer Treatment?	https://www.youtube.com/watch?v=ZKAe5cRILto
16	Natural Remedies for Skin Cancer: A Complementary Approach	https://www.youtube.com/watch?v=Mpa1qTyW6pE
17	Doctors Warn About The Use Of Black Salve As A Cure For Skin Cancer	https://www.youtube.com/watch?v=6L5Pbbj6ZEE
18	Basal Cell Carcinoma Treatment with Traditional and Alternative Remedies!	https://www.youtube.com/watch?v=9bdjYmz07BM
19	Top 4 natural remedies for skin cancer the FDA does NOT want you using: Part I	https://www.youtube.com/watch?v=byH3GH_g9lc
20	Natural Remedies For - Skin Toner - Skin Cancer - Hair Growth	https://www.youtube.com/watch?v=mbFUOREDyHw
21	Only 2 Spoons of Turmeric Powder Can Treatment Skin Cancer	https://www.youtube.com/watch?v=TFqtEBD5nv8
22	How to Treat Skin Cancer Using This Common Weed!	https://www.youtube.com/watch?v=2llivf5J-M0
23	Top 4 natural remedies for SKIN CANCER the FDA does NOT want you using: Part II	https://www.youtube.com/watch?v=cc53qyrgH0k
24	Protection From Skin Cancer and Natural Remedies	https://www.youtube.com/watch?v=_GAycsIYbbw
25	Natural Remedies	https://www.youtube.com/watch?v=IIFmuOHhU78
26	Natural Health Wound Care: Cancer	https://www.youtube.com/watch?v=MPzE9fAgq7o
27	Prevent and treat skin cancer with chayote, a powerful superfood	https://www.youtube.com/watch?v=heDTIS6krh4
28	Skin Cancer and Skin Lesions. Testimonies of Removal	https://www.youtube.com/watch?v=TkcB7N8uB2E
29	TOP 10 Natural Foods Proven to ANTI CANCER that You Must Eat Every Day | Christiansen Felix	https://www.youtube.com/watch?v=FGnWNUilZoY
30	6 Most Effective Herbal Remedies For Skin Cancer	https://www.youtube.com/watch?v=FBtwWPykaYA
31	HOW TO CURE SKIN CANCER NATURALLY	https://www.youtube.com/watch?v=PSzK98Macpg
32	HOW I'M HEALING SKIN CANCER NATURALLY (CAUSES, FOODS I EAT & PREVENTION)	https://www.youtube.com/watch?v=UJz7VjpG9M8
33	Natural Skin Cancer Treatment Methods	https://www.youtube.com/watch?v=mcApW8LT-N8
34	Top 6 Herbal Remedies For Skin Cancer	https://www.youtube.com/watch?v=L5pZdwafd8c
35	HOME REMEDIES FOR SKIN CANCER [ NATURAL HEALING]	https://www.youtube.com/watch?v=-7q_-Dt86k4
36	cure cancer naturally, cure cancer, heal cancer naturally, basal cell carcinoma, skin cancer	https://www.youtube.com/watch?v=-UrXggMlxlw
37	How I Healed My Mother’s Skin Cancer At Home	https://www.youtube.com/watch?v=ty_UOtKd2YQ
38	HOW I'M HEALING CANCER NATURALLY (3 BIG Life Changes)	https://www.youtube.com/watch?v=Nij4j1hkoKg
39	Herbs that can help fight cancer - How to cure cancer naturally and effectively	https://www.youtube.com/watch?v=33Rk3WiPPyE
40	H2O2 cure for skin cancer testimonial	https://www.youtube.com/watch?v=8q7esQFfzh8
41	Doctors Warn Of Dangerous Home Remedy That Touts Cancer Cure	https://www.youtube.com/watch?v=rDnSA3tNCAw&pp=ygUhYWx0ZXJuYXRpdmUgc2tpbiBjYW5jZXIgdHJlYXRtZW50
42	Natural Food Additive May Prevent Skin Cancer	https://www.youtube.com/watch?v=kql1WLLXnMs&pp=ygUhYWx0ZXJuYXRpdmUgc2tpbiBjYW5jZXIgdHJlYXRtZW50
43	Baking Soda and Coconut Oil: A Homeopathic Treatment for Skin Issues	https://www.youtube.com/watch?v=9qYbeUmQiik&pp=ygUhYWx0ZXJuYXRpdmUgc2tpbiBjYW5jZXIgdHJlYXRtZW50
44	2 Fruits Fight Cancer Naturally + Cancer-Fighting Drink Recipe (drink 2 tbps per day!)	https://www.youtube.com/watch?v=lUCjRsObPks&pp=ygUhYWx0ZXJuYXRpdmUgc2tpbiBjYW5jZXIgdHJlYXRtZW50
45	Demystified: Cancer, Supplements and Alternative Medicines	https://www.youtube.com/watch?v=rnh2N4OLi9E
46	Signs of Skin Cancer on Your Face	https://www.youtube.com/watch?v=cuuHTS7aVdg
47	Basal Cell Carcinoma: Causes and Natural Treatments ( Skin Cancer removers)	https://www.youtube.com/watch?v=n_JqglcZrDM
48	Alternative method for Treating Basal Cell Carcinoma, World Congress of Apitherapy, May 2021	https://www.youtube.com/watch?v=9DHHTI5mtck
49	Holistic Treatment of My Skin Cancer	https://www.youtube.com/watch?v=OLzwBbPsCw4
50	My journey in treating skin cancer and lymphoma using natural and alternative things that God made	https://www.youtube.com/watch?v=nyuB9n3N394
51	7 Amazing Herbal Remedies For Skin Cancer	https://www.youtube.com/watch?v=Yb1rQ668G68
52	Alternative Health: Ionized Water and Skin Cancer	https://www.youtube.com/watch?v=Q3L6UCPhFws
53	4 Natural Treatments For Skin Cancer	https://www.youtube.com/watch?v=0IfG5dvpHcA
54	Scabby nose skin cancer update... Petty Spurge, you beauty!	https://www.youtube.com/watch?v=5iF6mDGEgNw
55	Natural Help for Skin Cancer - Omega3 & Broccoli sprouts	https://www.youtube.com/watch?v=B3RnVOBI16A
56	Home Remedy #9 Skin Cancer Treatment	https://www.youtube.com/watch?v=JT_Eiwr72GQ
57	SUN PROTECTION - The Skin Cancer Fighting Foods Everyone Should Eat | Doctor ER	https://www.youtube.com/watch?v=35AsYTokma8&pp=ygUcbmF0dXJhbCBza2luIGNhbmNlciByZW1lZGllcw%3D%3D
58	Topical Application of Turmeric Curcumin for Cancer	https://www.youtube.com/watch?v=5YnmnPc6bFo
59	The Best Supplement to Prevent Skin Cancer	https://www.youtube.com/watch?v=Hv2OHfRtHC8
60	My Natural Skin Cancer Treatment	https://www.youtube.com/watch?v=ezUsvtRWN4c
61	How to Prevent Skin Cancer with Diet	https://www.youtube.com/watch?v=ovbc63gRT1A
